# Testing Implementation of FFC-AL-EIT

**DOI:** 10.1093/geroni/igab046.1321

**Published:** 2021-12-17

**Authors:** Barbara Resnick, Marie Boltz

## Abstract

Residents in assisted living settings engage in limited amounts of physical activity and decline functionally more rapidly than peers in nursing homes. To address the persistent functional decline and increased time spent in sedentary activity Function Focused Care was developed. Function Focused Care involves teaching caregivers to evaluate residents’ underlying functional capability and physical activity and engage them in physical activity during all care interactions. Prior research has demonstrated that implementing function focused care improves or maintains function and increases physical activity, improves mood and decreases behavioral symptoms among residents. To optimize implementation of Function Focused Care a theoretically based implementation strategy, Function Focused Care for Assisted Living Using the Evidence Integration Triangle (FFC-AL-EIT), was developed. FFC-AL-EIT combines the social ecological model, social cognitive theory and the Evidence Integration Triangle. The social ecological model includes intrapersonal, interpersonal, environmental, and policy factors that influence behavior. Social cognitive theory guides the interpersonal interactions that motivate caregivers and residents to engage in function focused care. Lastly, the Evidence Integration Triangle facilitates systemic implementation of function focused care. A total of 85 facilities from three states were randomized (FFC-AL-EIT versus Education Only) and 794 residents consented. The Reach, Effectiveness, Adoption, Implementation, and Maintenance (RE-AIM) model was used to evaluate outcomes. This symposium will provide the implementation outcomes and value of the Evidence Integration Triangle, the effectiveness of FFC-AL-EIT on function and physical activity and the effectiveness on psychosocial outcomes and care interactions.

